# Human Cytomegalovirus Replication Is Strictly Inhibited by siRNAs Targeting UL54, UL97 or UL122/123 Gene Transcripts

**DOI:** 10.1371/journal.pone.0097231

**Published:** 2014-06-02

**Authors:** Stuart T. Hamilton, Jens Milbradt, Manfred Marschall, William D. Rawlinson

**Affiliations:** 1 Virology Division, SEALS Microbiology, Prince of Wales Hospital, Sydney, Australia; 2 School of Biotechnology and Biomolecular Sciences, University of New South Wales, Sydney, Australia; 3 School of Medical Sciences, University of New South Wales, Sydney, Australia; 4 Institute for Clinical and Molecular Virology, University of Erlangen-Nuremberg, Erlangen, Germany; University of Regensburg, Germany

## Abstract

Human cytomegalovirus (HCMV) causes severe sequelae in immunocompromised hosts. Current antiviral therapies have serious adverse effects, with treatment in many clinical settings problematic, making new therapeutic approaches necessary. We examined the *in vitro* efficacy of small interfering RNAs (siRNAs) targeting the HCMV gene transcripts UL54 (DNA polymerase), UL97 (protein kinase) and UL122/123 (immediate-early proteins) as inhibitors of viral protein expression and virus replication in cell cultures. Two siRNAs for each HCMV target (designated A and B) were assessed for inhibition efficacy using western blot and standard plaque assays. Continuous human embryonic kidney 293T cells were treated with HCMV or non-specific scrambled (siSc) siRNA followed by transfection with plasmids expressing the target transcripts. Human MRC-5 fibroblasts were HCMV-siRNA or siSc treated, infected with HCMV strain AD169 (1 pfu/cell) and HCMV immediate-early (IE1p72 and IE2p86), early (pp65), early-late (pUL97) and true late (MCP) protein and virus progeny production measured during a single round of replication. Concordant results showed siUL54B, siUL97A and siUL122B displayed the most potent inhibitory effects with a reduction of 92.7%, 99.6% and 93.7% in plasmid protein expression, 65.9%, 58.1% and 64.8% in total HCMV protein expression and 97.2%, 96.2% and 94.3% (p<0.0001) in viral progeny production respectively. Analysing the siRNA inhibitory effects during multiple rounds of HCMV replication at a multiplicity of infection of 0.001 pfu/cell, siUL54B, siUL97A and siUL122B treatment resulted in a reduction of 80.0%, 59.6% and 84.5% in total HCMV protein expression, 52.9%, 49.2% and 58.3% in number of cells infected and 98.5%, 91.4% and 99.1% (p<0.0001) in viral progeny production at 7 dpi respectively. These results suggest potential *in vivo* siRNA therapies targeting the HCMV gene transcripts UL54, UL97 and UL122/123 would be highly effective, however, the antiviral efficacy of siRNAs targeting UL97 may be more highly dependent on viral load and methods of administration.

## Introduction

Human cytomegalovirus (HCMV) is a ubiquitous infection causing serious sequelae in immunocompromised patients and the developing fetus during pregnancy [1]. Since the global introduction of rubella immunisation programs, HCMV has become the leading infectious cause of congenital malformation in developed countries [2]. There are currently no licensed vaccines in routine use for the prevention of HCMV-induced disease. Any vaccine will take at least 20–30 years to reduce disease in pregnancy and immune compromise due to very high population seropositivity, and the importance of virus reactivation as the major cause of disease in transplant recipients and the fetus [3].

Few antiviral drugs are available for the treatment of HCMV infection - these include ganciclovir, valganciclovir, foscarnet, cidofovir, and fomivirsen. Treatment with these drugs is frequently associated with toxic side effects that make them unsuitable in some settings including during pregnancy. In immunocompromised patients, the emergence of drug resistant mutants is a significant problem in a subset of patients treated for long periods. There is therefore a current and pressing need for the development of novel drugs to treat HCMV infection, particularly those with reduced toxicity. Although novel developmental drugs such as letermovir (AiCuris, Wuppertal, Germany), which targets the viral DNA terminase complex [4]–[6], are presently undergoing clinical trials, some with promising results, final approval has not been achieved and the putative frequency of viral drug resistance has not been addressed in detail for most of these drugs.

Synthetic small interfering RNA (siRNA) sequences are potential therapeutic strategies for treatment of various pathologies including cancer, cardiovascular and neurological disease, and viral infection [7]. Recent *in vitro* studies have demonstrated the effectiveness of siRNA inhibition of viral infection including HCMV [8]–[14] human immunodeficiency virus-1 [15], hepatitis B virus [16], hepatitis C virus [17], poliovirus [18], human papillomavirus [19] and influenza virus [20]. These data, combined with recent developments in siRNA technology such as reduced toxicity, reduced immunostimulatory effects, reduced off-target effects, higher efficacies, increased stability, and design of specific siRNA nanoparticle delivery vehicles have all made siRNA molecules a more promising potential therapeutic to treat HCMV infection and disease [7].

During infection of permissive cells, HCMV undergoes temporally regulated, sequential gene expression [21]. Gene expression is initiated from the immediate-early region genes, which are transcribed by host cell RNA polymerase and expressed without requiring prior viral transcription [22]. HCMV immediate-early proteins include IE1p72 and IE2p86, which are produced by alternate splicing of a single precursor transcript (UL122/123), in addition to various auxiliary proteins [23]. The primary role of immediate-early genes is transcriptional transactivation of replication genes within the HCMV genome, with early and late gene expression being dependent upon immediate-early gene expression [21], [24], [25]. The antiviral drug fomivirisen is a 21-nucleotide anti-sense RNA that acts as a translational inhibitor of HCMV immediate-early mRNA which is an established second-line therapy for local treatment of HCMV retinitis. Early HCMV genes encode structural proteins necessary for viral production, in addition to proteins that are involved in viral DNA replication. These proteins include the viral DNA polymerase enzyme pUL54, an essential protein for HCMV replication, and viral protein kinase pUL97, which is non-essential but required for efficient HCMV replication. Both pUL54 and pUL97 are established antiviral targets for HCMV, with the former being the principal target for the majority of current antivirals. The late genes primarily encode structural and other proteins required for virus assembly, egress and other functions that enable virus reactivation and release [26], [27].

Previous publications [8]–[14] have examined siRNA anti-HCMV efficacies targeting the UL54, UL97 and UL122/123 transcripts; however, to date there has been no quantitative comparison of these three HCMV gene transcripts as targets in one identical system. We analysed the inhibitory efficacies of siRNAs targeting the HCMV transcripts UL54, UL97 and UL122/123 by measuring HCMV immediate-early (IE1p72 and IE2p86), early (pp65), early-late (pUL97) and true late (MCP) protein expression and virus progeny production during both single and multiple rounds of HCMV replication in MRC-5 fibroblasts. Results showed that treatment of fibroblast cells with HCMV siRNAs targeting UL54, UL97 and UL122/123 all had significant inhibitory effects on HCMV replication during both single and multi-round replication cycles, results which can be utilised for future *in vivo* studies.

## Results

### Different HCMV siRNAs Display Varying Efficacies at Inhibiting Plasmid-derived Expression of their Specific Gene Transcript Targets

**Figure 1:**
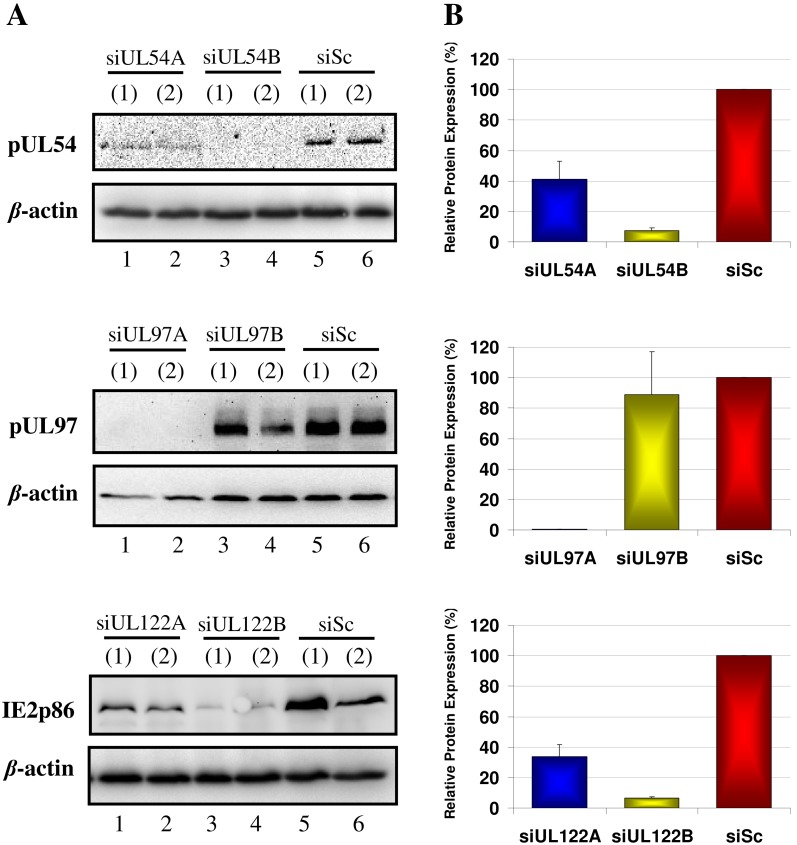
siRNA efficacy at inhibiting plasmid-derived HCMV protein expression. 293T cells were transfected with 50 µg of plasmid expressing the HCMV siRNA target transcript 24 hours post siRNA transfection. (A) Cells were harvested 72 hours post plasmid transfection and protein expression measured using western blot. (B) Protein expression in siRNA treated and plasmid transfected cells relative to cells treated with control scrambled siRNA (siSc) measured using densitometry. Results are from duplicate biological experiments and densitometry results presented as mean ± SD.

Two siRNA constructs were designed for each of the UL54, UL97 and UL122/123 HCMV transcript targets (designated as A and B) to find siRNA sequences with high inhibitory properties. The efficacies of each siRNA at silencing protein production were measured using western blot of the cell lysates of 293T cells treated with the HCMV siRNAs and transfected with plasmids expressing the specific HCMV proteins (pUL54, pUL97 and IE2p86 respectively), at 72 hr post plasmid transfection (Figure 1A). Densitometry was utilised to quantitate the siRNA inhibitory effects (Figure 1B). For UL54 siRNA silencing efficacies, siUL54A showed a modest reduction (58.7%) in pUL54 expression compared with cells transfected with scrambled siRNA (siSc), whereas siUL54B showed a substantial reduction (92.7%). The 293T cells treated with siUL97A showed an almost complete knockdown in pUL97 production (99.6%), whereas siUL97B showed little inhibitory effect (11.1%). For the UL122 siRNAs, siUL122A showed a modest reduction (66.4%) in IE2p86 production whereas siUL122B showed a substantial reduction (93.7%).

### siRNA Efficacy at Silencing Plasmid-derived HCMV Protein Expression Correlate with Efficacy at Inhibiting HCMV Protein Expression and Virus Progeny Production During a Single Round of Replication

**Figure 2:**
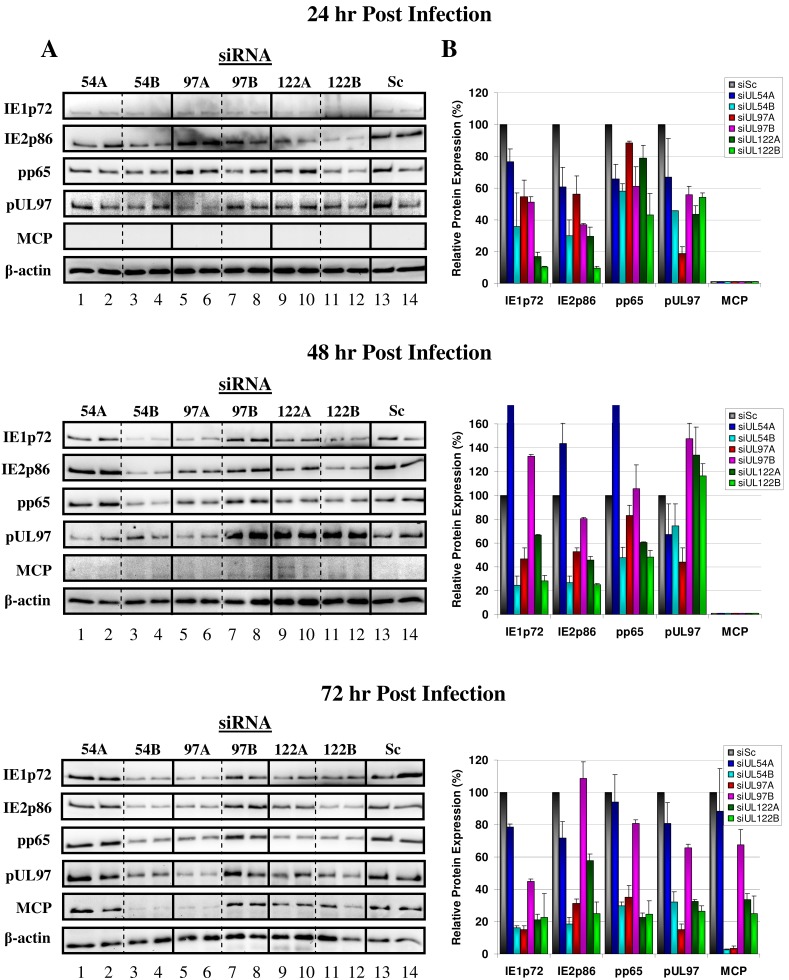
siRNA efficacy at inhibiting HCMV protein expression during a single round of HCMV replication at high multiplicity of infection. MRC-5 cells were transfected with 50 nM of non-specific scrambled control siRNA (siSc) or siRNAs targeting HCMV transcripts UL54 (siUL54A/B), UL97 (siUL97A/B), and UL122/123 (siUL122A/B) followed by infection with HCMV (1 pfu/cell) at 24 hours post transfection. (A) Cells were harvested 24, 48 and 72 hours post infection and HCMV immediate-early (IE1p72 and IE2p86), early (pp65), early-late (pUL97) and true late (MCP) protein expression measured using western blot. (B) Protein expression in UL54A/B, UL97A/B and UL122A/B siRNA treated and HCMV-infected cells relative to cells treated with scrambled siRNA (siSc) measured using densitometry. Results derived from two representative samples from triplicate biological experiments and densitometry results presented as mean ± SD.

MRC-5 fibroblasts were transfected with the siRNAs targeting UL54 (siUL54A and siUL54B), UL97 (siUL97A and siUL97B), UL122 (siUL122A and siUL122B) or siSc treated, followed by HCMV infection at a multiplicity of infection (MOI) of 1 pfu/cell at 24 hours post siRNA transfection. The efficacies of each siRNA at silencing HCMV immediate-early (IE1p72 and IE2p86), early (pp65), early-late (pUL97) and true late (MCP) protein production during a single round of replication were measured at 24, 48 and 72 hours post infection (hpi) using western blot (Figure 2A). Densitometry was used to quantitate the HCMV siRNA inhibitory effects relative to siSc treatment (Figure 2B). For UL54 silencing efficacies, siUL54A showed minimal or no inhibitory effects on HCMV protein production during the replication time course, and at 48 hpi resulted in accelerated IE1p72, IE2p86 and pp65 gene expression. The siUL54B siRNA however, consistently inhibited expression of all HCMV gene products during the replication cycle and showed the largest inhibitory effect on HCMV true late MCP of all siRNA treatments at 72 hpi (97.2%). Treatment with siUL97A also showed strong inhibitory effects on HCMV protein production, with inhibition of HCMV MCP production similar to siUL54B treatment at 72 hpi (96.7%) and displayed the strongest inhibitory effect on pUL97 expression (81.3% at 24 hpi). Treatment with siUL97B, which showed low efficacy in inhibiting pUL97 expression in the 293T/plasmid system, also showed low inhibitory effects on HCMV protein production with only a 32.5% reduction in MCP expression and 44.3% reduction in pUL97 expression 24 hpi. Cells treated with both UL122A and UL122B showed similar inhibitory effects however, siUL122B had the higher inhibitory effect over the three day time course. HCMV MCP production in response to siUL122A and siUL122B at 72 hpi was reduced by 66.3% and 75.2% respectively and showed the strongest inhibitory effect on IE1p72 (83.3% and 89.6% respectively) and IE2p86 (70.6% and 90.6% respectively) expression at 24 hpi. Analysing the total knockdown effect on all HCMV proteins during the three day time course revealed the largest knockdown was from siUL54B (65.9%) followed by siUL122B (64.8%), siUL97A (58.1%), siUL122A (50.5%), siUL97B (20.1%) and finally siUL54A (3.2%) (Data not shown).

**Figure 3:**
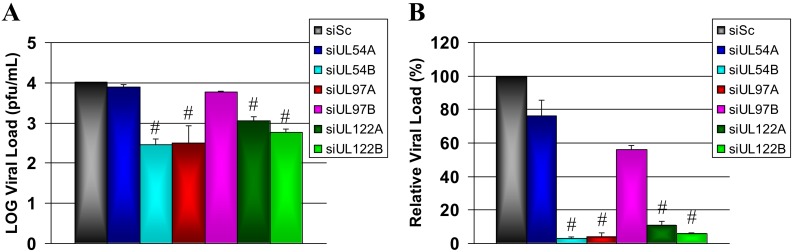
siRNA efficacy at inhibiting HCMV viral progeny production during a single round of HCMV replication at high multiplicity of infection. MRC-5 cells were transfected with 50 nM of non-specific scrambled control siRNA (siSc) or siRNAs targeting HCMV transcripts UL54 (siUL54A/B), UL97 (siUL97A/B), and UL122/123 (siUL122A/B) followed by infection with HCMV (1 pfu/cell) at 24 hours post transfection. (A) Supernatant was harvested 72 hours post infection and viral titre measured using plaque assay. (B) Viral load in HCMV-infected cells treated with siUL54A/B, siUL97A/B and siUL122A/B relative to HCMV-infected cells treated with scrambled siRNA (siSc). Data presented as mean of triplicate biological experiments ± SD. Significant differences between HCMV siRNA and siSc treated groups is given as #p<0.0001.

The observed siRNA inhibitory effect on HCMV protein production during single round replication was confirmed by performing plaque assays on cell culture supernatant 72 hpi to assess viral progeny production in response to the various siRNA treatments (Figure 3A). These plaque assays showed siRNA treatment inhibited viral progeny production after a single round of replication with varying efficacies and results were consistent with the siRNA effects observed on both plasmid- and HCMV-derived protein production. Treatment of cells with HCMV siRNAs reduced supernatant virus titre by 23.9% for siUL54A (p = 1.000), 97.2% for siUL54B (p*<*0.0001), 96.2% for siUL97A (p*<*0.0001), 44.2% for siUL97B (p = 1.000), 89.0% for siUL122A (p*<*0.0001) and 94.3% for siUL122B (p*<*0.0001) treated cells relative to siSc treated cells (Figure 3B).

### HCMV siRNAs Targeting UL54, UL97 and UL122/123 Gene Transcripts Inhibit HCMV Protein and Viral Progeny Production during Multiple Rounds of Replication

**Figure 4:**
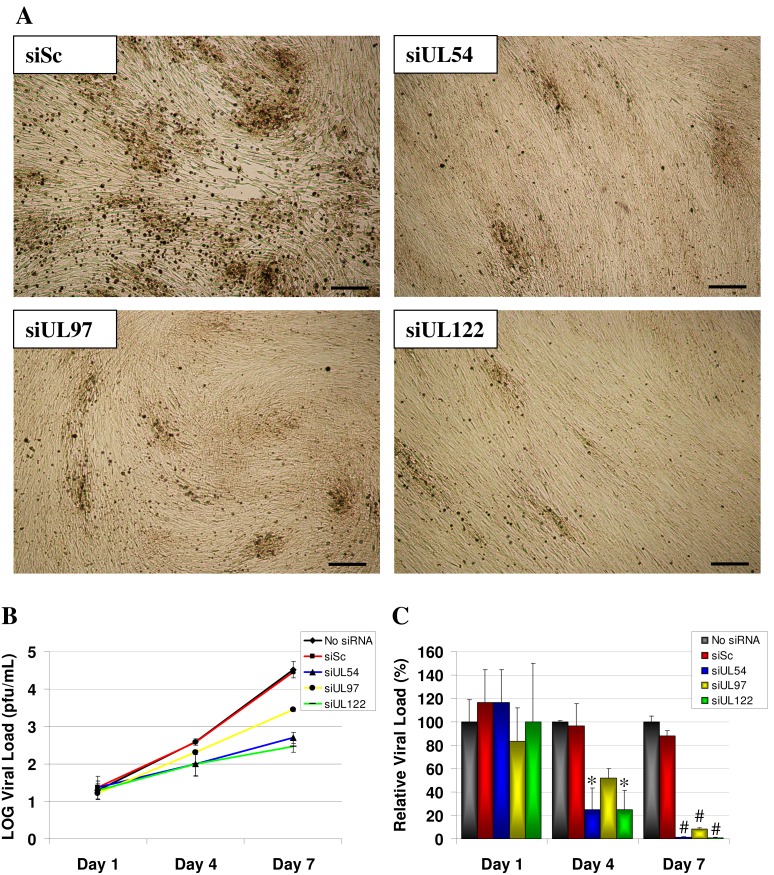
siRNA inhibition of HCMV replication and virus progeny production during multiple rounds of virus replication at low multiplicity of infection. (A) MRC-5 cells were untreated or transfected with 50 nM of non-specific scrambled control siRNA (siSc) or siRNAs targeting HCMV transcripts UL54 (siUL54B), UL97 (siUL97A), and UL122/123 (siUL122B) and infected with HCMV AD169 (0.001 pfu/cell) at 24 hours post siRNA transfection. Representative images of HCMV-induced CPE are shown at 7 days post infection in siRNA transfected cells. Scale bars represent 100 µm. (B) HCMV growth curves in response to siRNA treatment or no treatment with (C) viral load relative to no siRNA treatment. Data presented as mean of triplicate biological experiments ± SD. Significant differences between siRNA treated and untreated groups is given as *p<0.05 and #p<0.0001 and shown in (C).

The most inhibitory siRNAs for each HCMV gene transcript (siUL54B, siUL97A and siUL122B) were used to investigate their inhibitory effects on HCMV replication at lower multiplicities of infection (0.001 pfu/cell) and during multiple rounds of replication. The efficacy of siUL54B, siUL97A and siUL122B at inhibiting virus replication in MRC-5 fibroblasts was first evident by a reduction in viral cytopathic effect (CPE) (Figure 4A). At 7 days post infection (dpi), cells treated with siSc or no siRNA showed extensive CPE throughout the entire monolayer. However, cells treated with siUL54B, siUL97A and siUL122B showed limited CPE and modest plaque formation (Figure 4A). These results demonstrated the antiviral efficacy of siRNA treatment in limiting virus spread through the cell culture monolayer. The production of virus progeny was then measured using standard plaque assays on cell culture supernatant (Figure 4B and 4C). At 1 dpi, no significant difference in the amount of extracellular virus was observed between the siRNA treated and untreated groups (p = 1.000). By 4 dpi, a 75.0% reduction in supernatant virus progeny relative to untreated cells was observed in cells treated with siUL54B (p = 0.026) and siUL122B (p = 0.029) and a 47.9% reduction in siUL97A treated cells (p = 1.000). At 7 dpi, the inhibitory effect of the siRNAs on virus production had increased with siUL122B treatment (99.1%) compared with siUL54B (98.5%) and siUL97A (91.4%) treatment (p*<*0.0001). No inhibition of virus replication was observed in cells treated with siSc at any time point (p = 1.000). The alternative siRNA sequences that performed poorly at inhibiting protein expression in the 293T cell/plasmid system (siUL54A, siUL97B and siUL122A) also showed poor inhibitory effects on virus progeny production (data not shown).

### siRNA Inhibition of Virus Progeny Production at Low Multiplicities of Infection and Multiple Rounds of HCMV Replication was Concordant with Inhibition of Intracellular HCMV Protein Expression

**Figure 5:**
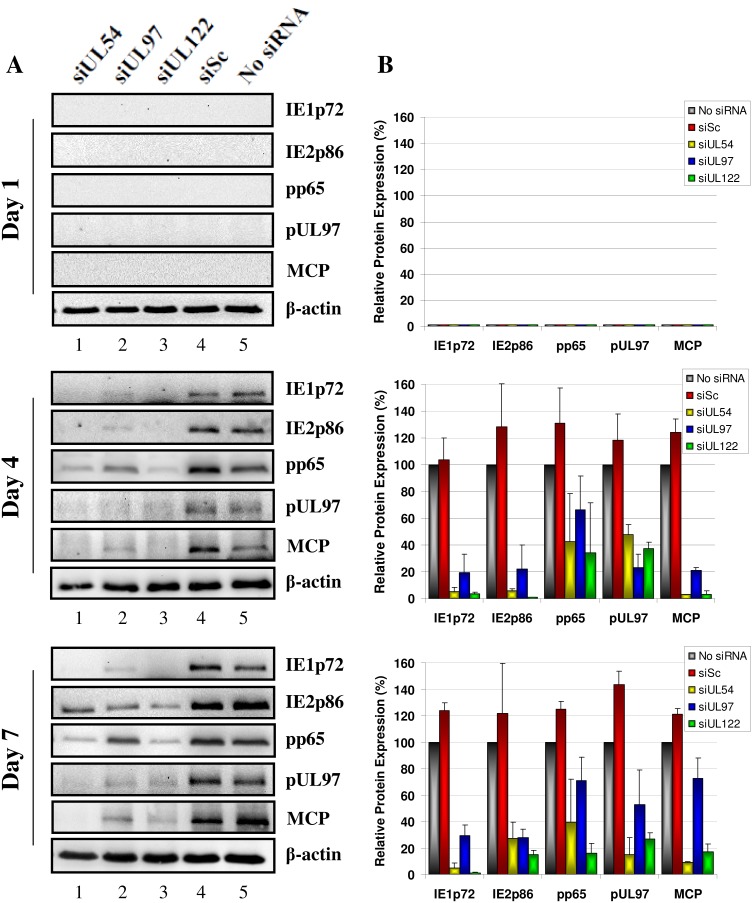
siRNA inhibition of HCMV protein expression during multiple rounds of viral replication at low multiplicity of infection. MRC-5 cells were untreated or transfected with 50 nM of non-specific scrambled control siRNA (siSc) or siRNAs targeting HCMV transcripts UL54 (siUL54B), UL97 (siUL97A), and UL122/123 (siUL122B) and infected with HCMV AD169 (0.001 pfu/cell) at 24 hours post siRNA transfection. (A) Cells were harvested 1, 4 and 7 days post infection and HCMV immediate-early (IE1p72 and IE2p86), early (pp65), early-late (pUL97) and true late (MCP) protein expression measured using western blot (B) Protein expression in HCMV-infected, siRNA treated cells relative to HCMV-infected, untreated cells measured using densitometry. Western blot images (A) are of one representative sample from triplicate experiments and densitometry data (B) presented as mean ± SD of triplicate biological experiments.

The production of intracellular HCMV immediate-early (IE1p72 and IE2p86), early, (pp65), early-late (pUL97) and true late (MCP) proteins in HCMV-infected MRC-5 cell lysate in response to siRNA treatment was assessed using western blot analysis (Figure 5A). Densitometry was utilised to quantitate the siRNA inhibitory effects relative to untreated cells (Figure 5B). These cell lysates were from the same treated cells used to examine virus-induced CPE and extracellular virus titres by plaque assay (Figure 2). HCMV proteins were not detected using western blot at 1 dpi, most probably due to the low MOI used (0.001 pfu/cell). At 4 dpi, treatment of cells with siUL54B reduced IE1p72, IE2p86, pp65, pUL97 and MCP expression by 94.8%, 94.1%, 57.6%, 51.9% and 97.1% respectively, siUL97A treatment by 80.6%, 78.1%, 33.6%, 77.0% and 79.1% respectively and siUL122B treatment by 96.4%, 99.1%, 65.6%, 62.4% and 97.1% respectively. By 7 dpi, treatment of cells with siUL54B reduced IE1p72, IE2p86, pp65, pUL97 and MCP expression by 95.1%, 73.0%, 60.8%, 85.1% and 90.7% respectively, siUL97A treatment by 70.9%, 72.2%, 29.2%, 47.2% and 27.7% respectively and siUL122B treatment by 99.1%, 85.3%, 84.0%, 73.5% and 82.9% respectively. Cells treated with siSc did not have reduced viral protein expression at any time point. Analysing the total knockdown effect on all HCMV proteins during the seven day time course revealed the most inhibitory siRNA was siUL122B (84.5%) followed by siUL54B (80.0%) and finally siUL97A (59.6%) (Data not shown).

**Figure 6:**
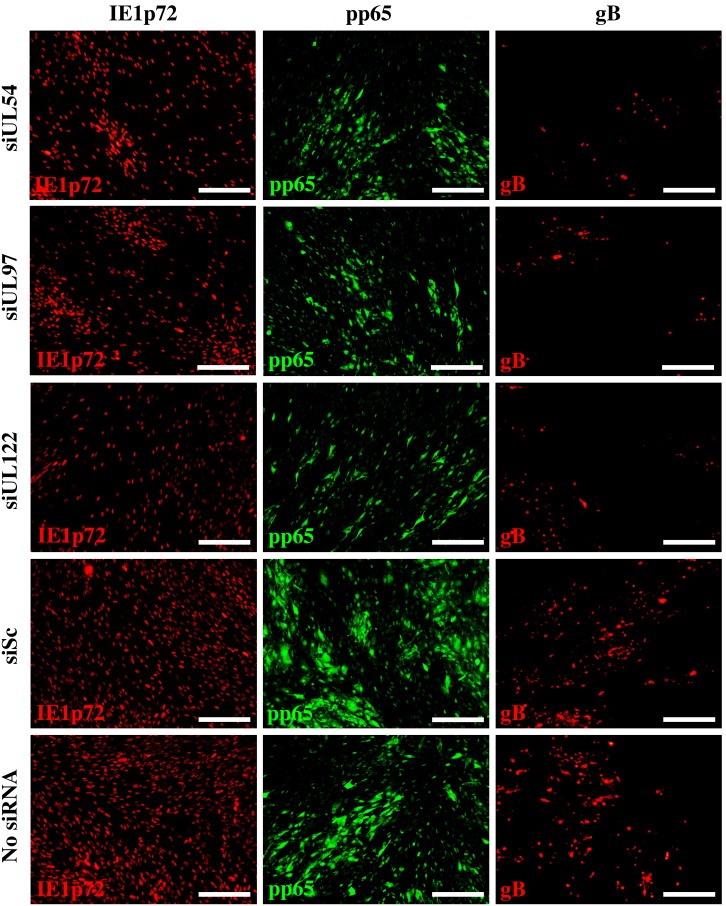
siRNA inhibition of HCMV dissemination and intracellular viral protein expression after multiple rounds of virus replication at low multiplicity of infection. MRC-5 cells were untreated or transfected with 50 nM of non-specific scrambled control siRNA (siSc) or siRNAs targeting HCMV transcripts UL54 (siUL54B), UL97 (siUL97A), and UL122/123 (siUL122B) and infected with HCMV AD169 (0.001 pfu/cell) at 24 hours post siRNA transfection. (A) Cells were harvested 1, 4 and 7 days post infection and HCMV immediate-early (IE1p72), early (pp65), and early-late (gB) protein expression detected using immunofluorescence. Representative images are of HCMV-infected, siRNA treated or untreated cells 7 days post infection. Scale bars represent 300 µm.

**Figure 7:**
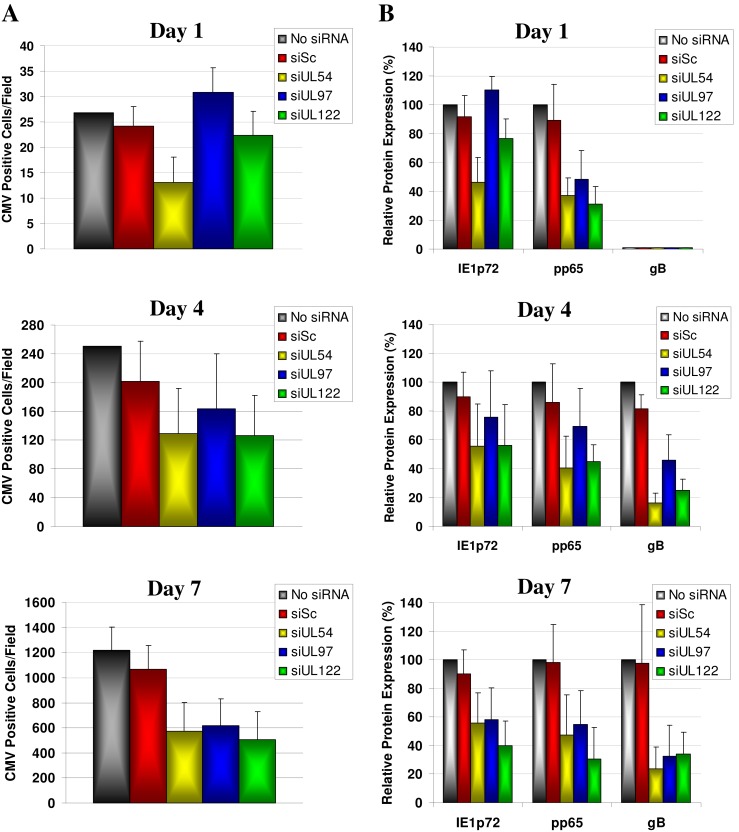
siRNA inhibition of HCMV dissemination and intracellular viral protein expression during multiple rounds of virus replication at low multiplicity of infection. MRC-5 cells were untreated or transfected with 50 nM of non-specific scrambled control siRNA (siSc) or siRNAs targeting HCMV transcripts UL54 (siUL54B), UL97 (siUL97A), and UL122/123 (siUL122B) and infected with HCMV AD169 (0.001 pfu/cell) at 24 hours post siRNA transfection. Cells were harvested 1, 4 and 7 days post infection and HCMV immediate-early (IE1p72), early (pp65), and early-late (gB) protein expression measured using immunofluorescence and quantitative image analysis. Data presented relative to HCMV-infected untreated cells and represent mean of 10 fields of view ± SD from duplicate biological experiments.

The HCMV protein expression kinetics in response to siRNA treatment were confirmed by immunofluorescence staining for HCMV immediate-early (IE1p72), early (pp65) and early-late (gB) proteins at 1, 4 and 7 dpi. Representative images of HCMV IE1p72, pp65 and gB protein expression at 7 dpi are shown in Figure 6. Quantitative image analysis showed UL54B, UL97A and UL122B HCMV siRNAs reduced the number of HCMV-infected cells by 52.9%, 49.2% and 58.3% respectively at 7 dpi relative to untreated cells (Figure 7A). This was determined by positive nuclear staining for IE1p72 protein, which is expressed during all stages of HCMV replication. HCMV protein expression was also inhibited over time with a reduction of IE1p72, pp65 and gB of 44.2%, 52.9% and 76.4% respectively for siUL54B treatment, 41.8%, 45.4% and 67.7% respectively for siUL97A treatment and 60.2%, 69.3% and 66.1% respectively for siUL122B treatment at 7 dpi (Figure 7B). No differences in number of HCMV-infected cells or differences in IE1p72, pp65 and gB protein expression in response to siSc treatment relative to no siRNA treatment were observed over the time course.

## Discussion

The two siRNAs designed for each of the HCMV gene transcripts UL54, UL97 and UL122/123 had different efficacies at inhibiting protein expression from specific plasmid constructs. They had concordant efficacies at inhibiting HCMV immediate-early (IE1p72 and IE2p86), early (pp65), early-late (pUL97) and true late (MCP) protein expression and viral progeny production in virus culture experiments. The MRC-5 fibroblasts transfected with the HCMV siRNAs and infected with HCMV at an MOI of 1 pfu/cell showed similar siRNA inhibitory efficacies at both the protein (Figure 2) and whole virus (Figure 3) level during a single round of replication. The siRNA most efficient at inhibiting HCMV virus progeny production at 72 hpi was siUL54B (97.2%, p<0.0001) followed by siUL97A (96.2%, p<0.0001), siUL122B (94.3%, p<0.0001), siUL122A (89.0%, p<0.0001), siUL97B (44.2%, p = 1.000) and finally siUL54A (23.9%, p = 1.000). The concordant efficacies of each individual siRNA in inhibiting their specific transcript targets in the 293T cell/plasmid system and HCMV protein expression and viral progeny production during a single round of viral replication strongly suggest siRNA inhibition was a specific effect of HCMV transcript knockdown and not a result of non-specific, off-target effects or sequence-independent siRNA activity. Although a putative impact of additional off-target effects (e.g. against cellular sequences) can never be ruled out completely, our controls with obviously unaffected expression of cellular housekeeping protein (β-actin) as well as monitoring of normal cell morphology and growth (microscopic inspection) renders this possibility rather unlikely.

To determine the siRNA efficacies at inhibiting HCMV growth over multiple rounds of replication, MRC-5 cells were transfected with the most efficient siRNAs for each target (siUL54B, siUL97A and siUL122B) and infected at a low MOI of 0.001 pfu/cell. The siRNA to HCMV transcript UL122/123 showed the greatest inhibitory effects on virus progeny production (99.1%, p<0.0001), followed by siUL54B (98.5%, p<0.0001) and finally siUL97A (95.7%, p<0.0001). Western blot and immunofluorescence analyses of intracellular HCMV protein production provided convergent data with the plaque assay analyses, showing siUL54B and siUL122B to be the most effective at blocking HCMV protein (IE1p72, IE2p86, pp65, pUL97, gB and MCP) production, while siUL97A was the least effective of the three siRNAs.

Why siUL97A showed potent anti-HCMV activity at the higher MOI of 1 pfu/cell during single round replication but did not display such strong activity at the lower MOI of 0.001 pfu/cell during multiple rounds of replication is unclear. The siUL97 multiplicity-dependent efficacy observed in this study may be attributed to pUL97 being a cyclin dependent kinase (CDK) ortholog with CDK-overlapping functions and activities [28], [29]. At the higher MOI of 1 pfu/cell, HCMV would arrest the cell cycle efficiently and simultaneously and thereby result in low level cellular CDK activity, so that pUL97 kinase activity would be more crucial for HCMV replication than during low MOI. However, at the lower MOI of 0.001 pfu/cell, cell cycle arrest would be less pronounced, therefore higher cellular CDK activity would be available to supplement an siRNA-mediated deficiency in pUL97 activity, resulting in only a modest level of antiviral response. This is consistent with evidence that the antiviral activity of the anti-HCMV antiviral maribavir, which also targets UL97, is influenced by culture conditions and metabolic status of host cells, particularly that inhibition of cellular CDKs potentiates the antiviral effects of maribavir [28], [30]. Interestingly, siUL97A treatment was also observed to inhibit HCMV IE1p72 expression during a single round of HCMV replication. This observation is consistent with the recent finding of Bigley *et al.* who showed the HCMV pUL97 kinase regulates HCMV immediate-early gene expression by phosphorylation-mediated disruption of histone deacetylase 1 binding to the major immediate-early promoter [31].

While there have been previous investigations into the efficacies of various siRNAs targeting the HCMV transcripts UL54 and UL122/123 at inhibiting HCMV replication *in vitro* [8], [10]–[14], there has only been one previous study to investigate the efficacy of siUL97 treatment [9]. Shin *et al.* [9] showed siUL97 was more effective than siUL54 at inhibiting HCMV mRNA (UL83) and protein (IE2p86 and pUL83) expression during a four day culture time course. However, this study did not examine the effect on production of infectious virus progeny in response to treatment, which makes comparisons to other siRNA studies difficult. Our data suggest that the presently analysed siRNA targeting the UL97 protein kinase transcript (siUL97A) displays potent anti-HCMV activity comparable to siRNAs targeting the essential HCMV transcripts UL54 and UL122/123. However, this effect is multiplicity dependent *in vitro*, with siUL97A losing substantial antiviral activity at lower multiplicities of infection. As pUL97 is a multifunctional protein kinase, it might possess crucial importance for HCMV replication and pathogenesis *in vivo* (reviewed by Marschall *et al.* [32]). In fact, pUL97 is a validated target for ongoing analyses of the developmental drug maribavir in clinical studies by ViroPharma. Thus, the moderate efficacy of UL97-directed siRNAs measured in the present study at low multiplicities of infection might possibly more reflect a limitation of this approach *in vitro* rather than a general lack of efficacy of UL97-directed siRNA sequences for *in vivo* applications.

Despite the demonstrated inhibitory efficacies of the siRNAs siUL54B and siUL122B targeting the HCMV essential gene transcripts UL54 and UL122/123, complete inhibition of virus replication was not achieved in this study. This limitation could be attributed to i) the lower transfection efficiencies for primary fibroblast cell cultures versus more transfection efficient cell lines such as 293T cells ii) the transient and single administration of synthetic siRNA molecules as opposed to continuous siRNA expression from vector systems containing shRNA iii) residual target gene expression resulting from less than 100% knockdown efficiency or iv) HCMV viral countermeasures against the RNAi pathway, as has been observed with other RNA and DNA viruses (reviewed in [33]). While the potential exists for HCMV viral resistance to siRNA therapy via mutations of the targeted region or virus-encoded suppressors of the siRNA pathway, this could be minimised by targeting highly conserved regions of the target transcript and/or using a combined therapy of siRNAs targeting both conserved regions of a target transcript and siRNAs targeting any virus-encoded siRNA pathway suppressors. Furthermore, given the genomes of DNA viruses have a lower mutation rate (10^−6^ to 10^−8^ per incorporated nucleotide) than RNA viruses (10^−4^ to 10^−6^ per incorporated nucleotide) [34], finding highly conserved target sequences with minimal mutation risks would be much more feasible compared with RNA viruses such as HIV which have been shown to develop siRNA resistance via escape mutations readily [35]. However, recent evidence indicates HCMV intra-host genomic variability is comparable to many RNA viruses, possibly due to selective sweeps from immune-mediated mechanisms, emphasising the importance of targeting conserved sequences in future *in vivo* studies [36], [37].

The efficacy of siRNA molecules targeting HCMV transcripts has been demonstrated to inhibit HCMV replication in cell culture models. This is the first study to demonstrate differing efficacies between three siRNA transcript and antiviral protein targets in one system and at differing multiplicities of infection and rounds of replication. Our results indicate future design of siRNA therapeutics to inhibit HCMV infection *in vivo* could benefit from including both essential and non-essential HCMV gene transcript targets such as UL54, UL97 and UL122/123 which show strong and reproducible antiviral properties in cell culture systems.

## Methods

### Cell Lines and Preparation of Virus Stocks

Human MRC-5 fetal lung fibroblasts (ECACC) were maintained in Modified Minimum Essential Medium (MEM; Invitrogen) supplemented with 10% fetal bovine serum (FBS; Bovogen) and 100 U/ml penicillin G, 100 µg/ml streptomycin and 29.2 µg/ml L-glutamine (1X PSG, Invitrogen). Human embryonic kidney 293T cells were maintained in DMEM/F12+Gluta-MAX (Invitrogen) supplemented with 10% FBS and 1X PSG. Cell lines were demonstrated *Mycoplasma* free and maintained at 37°C with 5% CO_2_. The HCMV laboratory strain AD169 (ATCC) was propagated in MRC-5 cells maintained in MEM supplemented with 2% FBS and 1X PSG. Supernatants containing extracellular virus were stored at −80°C. The titre of virus stocks was determined using standard plaque assay in MRC-5 cells.

### siRNA Antisense Sequences

The siRNA antisense sequences used in this study were: siUL54A: 5′-AGGAC AGGUCAUCCUUGCGUU-3′, siUL54B: 5′- AUAGUUGGGCGAGUUAGUCUU-3′, siUL97A: 5′-GACACUGGUGAUUGAGAAAUU-3′, siUL97B: 5′-GUCCACGGCAUAACAGAUCUU-3′, siUL122A: 5′-UGACCUGUUUGGGAAACUUUU-3′, siUL122B: 5′-AAGACGAAGAGGAA CUAUCUU-3′.

### siRNA Inhibition Efficiency Assays

The 293T cells were seeded in 24 well plates and cultured to ∼40% confluence in DMEM/F12+Gluta-MAX supplemented with 10% FBS and 1X PSG. Cells were washed with PBS and media replaced with Opti-MEM without supplements. The custom designed siRNAs (Life Technologies) targeting HCMV UL54 (siUL54A and siUL54B), UL97 (siUL97A and siUL97B) and UL122/123 (siUL122A and siUL122B) transcripts were transfected at 50 nM concentrations using Lipofectamine 2000 (Life Technologies) following manufacturer’s protocols. Cells transfected with non-specific scrambled siRNA (siSc) (Life Technologies) at 50 nM concentrations served as negative controls. Following six hour incubation with the Lipofectamine/siRNA complexes, cell media was supplemented with 2% FBS and 1X PSG and cells incubated for a further 18 hours. Cells were again washed with PBS and media replaced with Opti-MEM. Plasmids expressing the HCMV viral proteins pUL97 (Marschall *et al*., 2001), pUL54 and IE2p86 (plasmid repository of the laboratories Manfred Marschall and Thomas Stamminger, Erlangen, Germany) were transfected (1 µg) into the 293T cells using Lipofectamine 2000 following manufacturer’s protocol. Following six hour incubation, cell media was replaced with DMEM/F12+Gluta-MAX supplemented with 10% FBS and 1X PSG and cells incubated for a further 72 hours. Cells were then harvested using 0.25% trypsin (Invitrogen) for western blot analyses.

### siRNA Transfection And Infection

MRC-5 cells were seeded in 24 well plates and cultured to ∼60% confluence in MEM supplemented with 10% FBS and 1X PSG. Cells were transfected with siUL54A/B, siUL97A/B, siUL122A/B, siSc or no siRNA as described above. 24 hours post transfection cells were inoculated with HCMV AD169 at multiplicity of infection of 1 pfu/cell or 0.001 pfu/cell in triplicate for each siRNA treated and untreated group. Plates were centrifuged at 770 x g for 30 min followed by 1 hr incubation at 37°C with 5% CO_2_. Supernatant was removed and replaced with OPTI-MEM supplemented with 2% FBS and 1X PSG. Cells were incubated at 37°C with 5% CO_2_ until cell harvest at days 1, 2, 3, 4, or 7 post infection.

### Viral Replication Curves

Total cell culture supernatant (1 ml) was harvested at specified time points and frozen at −80°C. Viral titres were determined on MRC-5 fibroblasts using standard plaque assay techniques. Plaques were counted at seven days post infection, and the titres for each triplicate biological siRNA experiment combined to give mean ± SD.

### Western Blot Analysis

Immediately following cell culture supernatant harvest, cells were washed with phosphate buffered saline (PBS) and harvested using 0.25% trypsin. Cells were washed with PBS and lysed with 2X SDS sample buffer (62.5 mM Tris/HCl (pH 6.8), 1 mM EDTA, 10% glycerol, 2% SDS, 5% β-mercaptoethanol, 0.005% bromophenol blue) at 95°C for 10 min followed by a brief vortex. Protein extracts were subjected to SDS-PAGE followed by transfer to a Protran nitrocellulose membrane (Whatman). Immunostaining was performed with the antibodies mAb-β-actin (Ac-15, Sigma), mAb-Flag (M2, Sigma), mAb-IE1p72/pUL44 (Clones DDG9 and CCH2; Dako), mAb-IE2p86 (Santa Cruz), mAb-pp65 (Abcam), mAb-pUL97 (#8, kindly provided by Detlef Michel, Ulm, Germany), mAb-MCP (major capsid protein; 28-4, kindly provided by William Britt, Birmingham, AL, USA**)** and HRP-conjugated anti-mouse secondary antibody (Pierce). Protein bands were visualised using chemiluminescence. Densitometry of immunostaining was performed using ImageJ software. The mean densitometry values for control infected-cells were assumed to be 100% and this value was used to calculate relative protein expression in HCMV-infected cells treated with HCMV siRNAs with results presented as mean ± SD of duplicate or triplicate biological experiments.

### Immunofluorescence

MRC-5 cells were seeded in 6 well plates with underlying coverslips and transfected with siRNAs followed by HCMV infection 24 hours post transfection at an MOI of 0.001 pfu/cell as described above. Immunofluorescence staining was performed at 1, 4 and 7 dpi as previously described [38]. Staining was performed with mAb-IE1p72/pUL44 (Clones DDG9 and CCH2; Dako), mAb-pp65 (Abcam), and mAb-gB (Abcam) primary antibodies and Alexa Fluor 488 and 594 goat anti-mouse secondary antibodies. For image analysis, 10 fields of view were captured for each treatment, immunostain and time point at x100 magnification. The percentage of positive pixels in each field of view was then calculated using ImageJ software. The mean positive pixel count for all 10 fields of view in untreated HCMV infected cells was assumed to be 100% and this value used to calculate relative protein expression in HCMV-infected cells treated with HCMV siRNAs. Data presented as mean ± SD.

### Statistical Analysis

For statistical analysis, HCMV-positive foci counts from plaque assay analyses were log transformed and differences between groups assessed using one way ANOVA with Bonferroni’s correction applied post hoc to adjust for multiple comparisons. Data were analysed using SPSS Statistics package (Version 21, IBM Corporation).
